# Polarization‐Controlled Dual‐Programmable Metasurfaces

**DOI:** 10.1002/advs.201903382

**Published:** 2020-04-16

**Authors:** Xin Ge Zhang, Qian Yu, Wei Xiang Jiang, Ya Lun Sun, Lin Bai, Qiang Wang, Cheng‐Wei Qiu, Tie Jun Cui

**Affiliations:** ^1^ State Key Laboratory of Millimeter Waves School of Information Science and Engineering Southeast University Nanjing 210096 China; ^2^ Department of Electrical and Computer Engineering National University of Singapore Singapore 119620 Singapore

**Keywords:** beam controls, digital logic platforms, dual‐programmable metasurfaces, orthogonally‐polarized electromagnetic waves, modular control circuits

## Abstract

Programmable metasurfaces allow dynamic and real‐time control of electromagnetic (EM) waves in subwavelength resolution, holding extraordinary potentials to establish meta‐systems. Achieving independent and real‐time controls of orthogonally‐polarized EM waves via the programmable metasurface is attractive for many applications, but remains considerably challenging. Here, a polarization‐controlled dual‐programmable metasurface (PDPM) with modular control circuits is proposed, which enables a dibit encoding capability in modifying the phase profiles of *x*‐ and *y*‐polarized waves individually. The constructed extended interface circuit is able to extend the number of control interfaces from a field programmable gate array by orders of magnitude and also possesses memory function, which enhance hugely the rewritability, scalability, reliability, and stability of PDPM. As a proof‐of‐concept, a wave‐based exclusive‐OR logic gate platform for spin control of circularly‐polarized waves, a fixed‐frequency wide‐angle dual‐beam scanning system, and a dual‐polarized shared‐aperture antenna are demonstrated using a single PDPM. The proposed PDPM opens up avenues for realizing more advanced and integrated multifunctional devices and systems that have two independent polarization‐controlled signal channels, which may find many applications in future‐oriented intelligent communication, imaging, and computing technologies.

## Graphical abstract

A polarization‐controlled dual‐programmable metasurface that can independently control orthogonally‐polarized microwaves in real time is proposed and realized experimentally. The dual‐programmable metasurface can achieve a lot of independent control channels via only one field‐programmable gate array based on the designed extensional interface circuit, thus providing attractive functions, including wave‐based exclusive‐OR logic operation for spin control, wide‐angle dual‐beam scanning, and dual‐polarized aperture sharing.

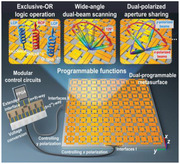

Metasurfaces, 2D equivalents of metamaterials, have demonstrated their superior abilities to realize full control of electromagnetic (EM) waves at the deep‐subwavelength thickness interface. As an emerging platform, metasurface has unlocked multifarious functional devices, such as flat lens,^[^ ^1^ ^]^ beam controller,^[^ ^2^, ^3^ ^]^ absorber and cloak,^[^ ^4^, ^5^ ^]^ hologram and imager,^[^ ^6^, ^7^ ^]^ and others.^[^ ^8^, ^9^, ^10^, ^11^, ^12^, ^13^ ^]^ In particular, active metasurfaces are capable of generating dynamically different functions as desired under the external stimulus. So far, many attempts have been made to exploit dynamic metasurfaces,^[^ ^14^, ^15^, ^16^, ^17^, ^18^, ^19^, ^20^ ^]^ however, most demonstrated dynamic metasurfaces are just tunable or reconfigurable, which only allow a fine‐tuning of similar functions or achieving several limited different functions, suffering poor rewritability. To realize real‐time control and switching of various functions, a field programmable gate array (FPGA) is introduced into dynamic metasurfaces to further construct programmable metasurfaces.^[^ ^21^ ^]^ Owing to programmable and real‐time manipulations of the EM waves, the digital coding and programmable metasurfaces have been investigated widely in the last few years and even extended to acoustic fields, ranging from functional verifications to device designs and system applications.^[^ ^6^, ^7^, ^21^, ^22^, ^23^, ^24^, ^25^, ^26^, ^27^, ^28^, ^29^ ^]^ However, in current programmable metasurfaces designs, the phase profiles of orthogonally‐polarized EM waves are coupled to each other or the programmability can only be achieved under a special polarization. Consequently, the single‐polarized programmable metasurfaces can only provide an effective information transmission channel to process multiple tasks serially. Moreover, most programmable metasurfaces to date just have a few independent control channels because of a limited number of control interfaces that one FPGA can provide, and thus they are adopted typically to realize some easy‐to‐implement functions. Both of these limitations greatly impair the programmability of the programmable metasurfaces, making it hard to achieve complex EM functions, and also severely restricting their ability to perform multiple tasks in parallel.

To improve the information processing efficiency and multitasking capability of metasurfaces, various approaches have been proposed to develop the dual‐polarized and chiral metasurfaces that enable independent controls of EM waves with different polarizations, and then provide two independent information channels in parallel.^[^ ^30^, ^31^, ^32^, ^33^, ^34^ ^]^ Compared with single‐polarized metasurfaces, the dual‐polarized metasurfaces can implement more complicated and attractive functions. Nevertheless, the existing dual‐polarized metasurfaces are just static or tunable, which cannot be controlled programmatically in real time, thus hampering their applications in high‐speed scanning systems and communications. Indeed, dual‐polarized programmable metasurfaces that allow real‐time and independent controls of two orthogonal polarizations are especially desirable in some important applications, such as the real‐time multichannel information processing, ultrafast polarization controls, and modulations, polarization‐division multiplexing, and dynamic dual‐polarized antennas and radar systems, but have so far remained elusive.

In this communication, we propose the concept and realize the prototype of a polarization‐controlled dual‐programmable metasurface (PDPM) for independent manipulations of orthogonally‐polarized microwaves in real time. Importantly, the PDPM can achieve a large number of independent control channels via only one FPGA by leveraging the designed extended interface circuit that can extend the interfaces of the FPGA by orders of magnitude. Accordingly, the realized PDPM possesses abundant programmability, and thus can provide more remarkable and more complex EM functions based on one physical configuration.

As depicted schematically in **Figure** **1**, the proposed PDPM has two sets of control interfaces (I and II) that can be driven independently by the specially designed modular control circuits. The extended interface circuit is able to extend the (*m* + 9)‐way control signals from FPGA to (8 × 2*^m^*)‐way control signals, which enormously increases the number of available control interfaces. Therefore, we can use one FPGA to provide a huge amount of independent control channels to actuate the PDPM in real time. The two sets of control interfaces are isolated from each other, which are used to receive two independent coding sequences from the FPGA. By changing the two coding sequences of PDPM, the phase profiles of *x*‐ and *y*‐polarized EM waves can be controlled individually in a programmable fashion, which provides two‐channel programmability independently. The dual‐programmable feature makes the proposed PDPM have remarkable and diverse capabilities for realizing attractive functionalities including wave‐based exclusive‐OR (XOR) logic operation for spin control, wide‐angle dual‐beam scanning, dual‐polarized aperture sharing, and so on (Figure 1), which are hard to achieve using single‐polarized programmable metasurfaces only with several control channels. Because of memory and extensible interface features of the control circuits, the proposed PDPM has high reliability, stability, and scalability, which could push the programmable metasurfaces one step closer towards more complicated and practical applications.

**Figure 1:**
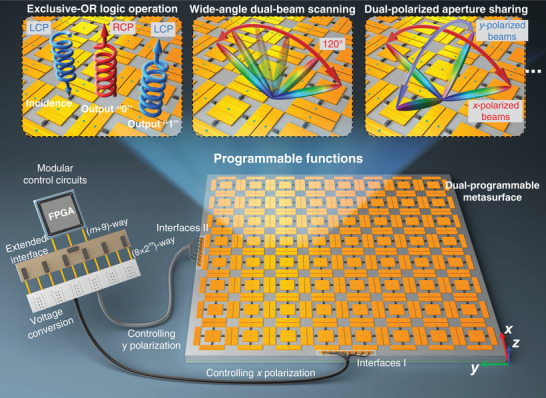
Proposed PDPM for real‐time and independent control of orthogonal‐polarized EM waves. Two sets of control interfaces I and II of the PDPM can be programmed independently by the specially designed modular control circuits for manipulating *x*‐ and *y*‐polarized EM waves, respectively. The realized PDPM has rich rewritability and high scalability, and can achieve versatile attractive functionalities, such as wave‐based XOR logic operation for spin control, fixed‐frequency wide‐angle dual‐beam scanning, dual‐polarized aperture sharing, and so on.

To construct PDPM, we first elaborately design a programmable metasurface element (ME) with the capability of manipulating two orthogonal‐polarized EM waves independently, as shown in **Figure** **2**a. The proposed ME contains a subwavelength resonator integrated with a direct current (DC) bias network. The resonator has two copper layers and a F4B dielectric layer (*ε*_r_ = 2.65, tan *δ* = 0.001). To realize the decoupled tuning of resonance states of the resonator under orthogonally polarized wave incidences, we devise a specific top metal pattern. The metal pattern is composed of a square copper patch and four identically rectangular copper patches that surround the square patch. The bottom of the substrate is a metal ground to prevent the transmission of EM waves. The two gaps in the *x*‐ and *y*‐directions can provide the equivalent capacitances of the resonator under the *x*‐ and *y*‐polarized incidences, respectively. To further achieve tunable capacitance, we integrate four identical varactors in the four gaps. With this designed topology, the equivalent capacitance of the resonator can be tuned independently under the *x*‐ and *y*‐polarized incidences through changing the capacitances of two varactors in the *x*‐ and *y*‐directions, respectively. Thus, when the *x*‐ and *y*‐polarized incident waves are projected onto the designed resonator individually, the resonator will exhibit different resonance states, thus generating two different phase profiles for the orthogonal polarization states.

**Figure 2:**
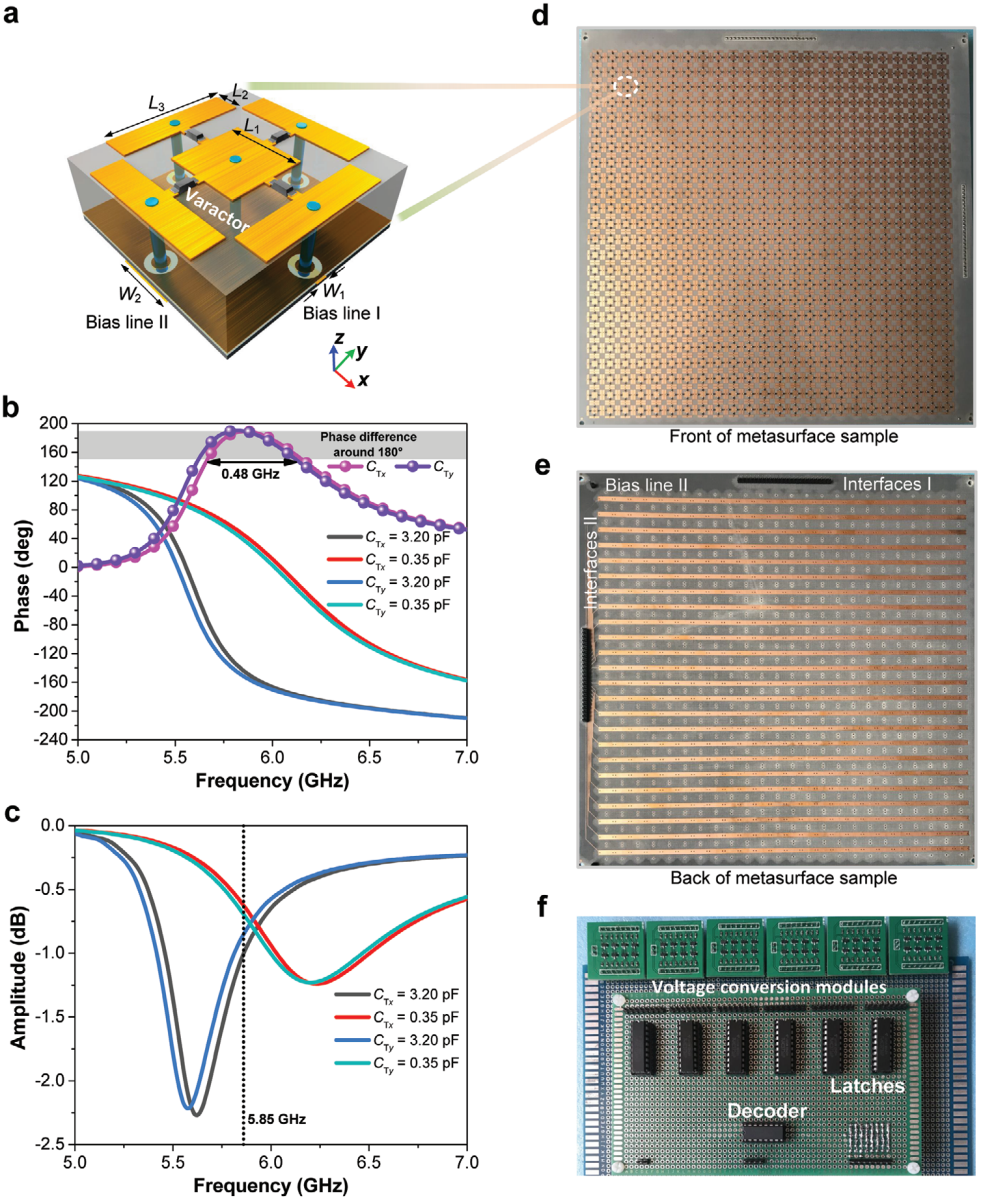
Designed dual‐programmable ME and fabricated prototype. a) 3D topological structure of the ME, in which the two pairs of varactors in the *x*‐ and *y*‐directions can be actuated independently by two bias lines I and II, respectively. b,c) Simulated phase responses and reflection amplitudes of the ME, respectively, for different capacitance values under the *x*‐ and *y*‐polarized incidences. The two phase differences are virtually identical ranging from 5.67 to 6.15 GHz and the reflection amplitudes are larger than −1 dB at 5.85 GHz. d,e) Front and back of the fabricated metasurface sample, respectively. f) Fabricated extended interface and DC voltage conversion circuits.

Because four varactors are embedded in the resonator, we design a DC bias network to provide reverse biases for the two pairs of varactors independently (see bias network design of ME in Supporting Information). Under this bias control, the two bias lines I and II can be controlled independently, and the two variodes in one direction are actuated simultaneously by one bias line, thus having the same capacitance. Because four varactors are integrated in one resonator, the effective reactance of the resonator is mainly determined by the features of the varactor. Thus, the key of our design is to choose varactors with suitable characteristics to realize the required resonance response. We finally use “Skyworks SMV2020‐079LF” varactor chip due to the low capacitance, low resistance, and high capacitance ratio at low reverse voltage. The variable capacitance *C*_T_ of the varactor can be tuned from 3.20 to 0.35 pF (capacitance ratio is approximately 9.14) under the reverse bias varying from 0 to 20 V.^[^ ^35^ ^]^ In the following design, variable *C*_T_ *_x_* represents the capacitance value of the two varactors in the *x*‐direction, and variable *C*_T_ *_y_* indicates the capacitance value of the varactors in the *y*‐direction.

We next investigate and analyze the reflection property of the designed ME using numerical simulation technology. See Supporting Information for more details on the structure parameters of the ME. The simulated reflection phase curves of the designed ME are plotted in Figure 2b for different capacitance configurations under the *x*‐ and *y*‐polarized incidences. When the capacitance vaule *C*_T_ *_x_* is tuned from 3.20 to 0.35 pF, we see clearly that the phase difference ranging from 150° to 188° can be achieved between 5.67 and 6.18 GHz for the *x*‐polarized EM waves, and when the capacitance vaule *C*_T_ *_y_* is tuned from 3.20 to 0.35 pF, the phase difference ranges from 150° to 190° between 5.62 and 6.15 GHz for the *y*‐polarized waves. We remark that, when the *x*‐ or *y*‐polarized wave propagates normally into the ME, changing the capacitance of the two varactors in the cross‐polarization direction has no effect on the resonance response (see Figure S2, Supporting Information), which indicates that the realized ME has high polarization stability and cross‐polarization isolation. Since the DC bias layer still has a thickness of 0.25 mm, the reflection phase curves of the ME are slightly different under the *x*‐ and *y*‐polarized incidences, despite the fact that *C*_T_ *_x_* = *C*_T_ *_y_*. However, in an operating frequency band ranging from 5.67 to 6.15 GHz, the two phase difference curves are nearly identical under the *x*‐ and *y*‐polarizations, and the phase differences are all 184° at 5.85 GHz. Moreover, the reflection amplitudes of the ME for different capacitance values are all larger than −2 dB in the working frequency band, and can reach above −1 dB at 5.85 GHz, as shown in Figure 2c. Hence, the designed programmable ME is able to realize “0” and “1” elements in 1‐bit digital coding metasurfaces through switching the capacitance of the loaded varactors dynamically. We encode the ME with *C*_T_ = 3.20 pF as the “0” element and that with *C*_T_ = 0.35 pF as the “1” element.

Based on the realized digital ME, we further design a 1‐bit PDPM to enable independent controls of two orthogonally linearly‐polarized EM waves in real time. The realized PDPM is an array of periodically‐arranged programmable MEs, which also contains two sets of control interfaces I and II that are connected to the control circuits (Figure 1). Each set of interfaces has multiple independent input pins, and every input pin is connected to a DC bias line for driving a string of digital elements simultaneously. To quickly program the metasurface, we propose and construct the modular control circuits that consist of an FPGA controller, an extended interface circuit with memory function, a DC voltage conversion circuit, and the corresponding program codes. The FPGA controller can output different control signals under driving the control programs. Generally, the programmable metasurfaces can be controlled by the FPGA output pins directly. However, the number of FPGA pins are commonly not many enough for controlling the programmable metasurfaces that need a mass of control inputs (e.g., metasurface with strong programmability and large‐scale metasurface). On the other hand, when metasurfaces are connected directly to FPGA, the functions of metasurfaces will be destroyed once the FPGA controller breaks down, such as FPGA power‐down, program error, and connection break‐off. To address these two problems, here we construct an extended interface circuit by employing decoders and a latch array. The proposed function‐extended interface circuit can not only expand the few numbers of FPGA pins to realize large‐scale control, but also maintain the original output state even if the connection of FPGA is disconnected. In this way, when the control of FPGA is interrupted, the current functionality of PDPM will not change, leading to a memory feature of the metasurface. The designed DC voltage conversion circuit is used to convert the low pin‐voltage of the extended interface circuit to the required 20 V voltage for the selected varactors.

As a demonstration example, we fabricate a PDPM prototype containing 24 × 24 elements and 48 independent input pins, as shown in Figures 2d,e. The 24 input pins of interface I are connected to 24 bias lines I for controlling the phase profile (varying along the *y*‐direction) of the *x*‐polarized EM waves, while the 24 input pins of interface II are connected to 24 bias lines II for controlling the phase profile (varying along the *x*‐direction) of the *y*‐polarized waves. Therefore, the proposed PDPM has rich rewritability and can be controlled independently in both directions. Figure 2f presents a photograph of the fabricated extended interface and DC voltage conversion circuits. Since each latch has 8 output pins, we use six latch chips to provide 48‐way control signals. The DC voltage conversion circuit contains six designed voltage conversion modules, each of which has 8‐way voltage conversion channels. Each DC voltage conversion module is actuated by a latch chip. See Experimental Section for more details on the design of the extended interface and voltage conversion circuits. The fabricated high‐speed interface circuit is able to expand 12‐way signals from the FPGA controller to 48‐way independent control signals. More importantly, the extended interface circuit has strong expansibility, which enables to realize very large‐scale controls through further increasing the number of address signals and latch chips. If we use *m*‐way address signals to drive corresponding multiple decoders, the (*m* + 9)‐way control signals from FPGA can be extended exponentially to (8 × 2*^m^*)‐way control signals. For example, by adopting the proposed extended interface circuit, in theory the 20‐channel FPGA is able to provide 16 384‐way control signals, which originally requires 820 such FPGA controllers to achieve.

Since each programmable ME has only two digital states of “0” and “1”, the metasurface can be programmed effectively by switching different coding sequences controlled by the integrated circuit module. Hence the proposed PDPM is capable of performing various tasks. We first evaluate the efficiency of designed metasurface at working frequency 5.85 GHz. Under the *x*‐ and *y*‐polarized incidences, when all MEs are in “0” state, the efficiencies of the metasurface are 81.2% in simulation and 76.8% in experiment; when all MEs are in “1” state, the efficiencies are 84.6% in simulation and 79.5% in experiment, respectively. Next, we demonstrate three advanced electronic systems based on the same PDPM prototype: a wave‐based dynamic XOR logic gate platform, a dual‐beam scanning antenna system, and a dual‐polarized shared‐aperture antenna system.

In PDPM, two opposite EM responses of the ME are characterized by digital “0” and “1”. Thus the constructed PDPM enables wave‐based information coding and processing on the physical level. Since the designed PDPM possesses two set of independently‐actuated electric input interfaces, it is able to realize two‐input logic operations. In this case, the 24 input pins in each set of interfaces have the same state to mimic an electric input. In physics, spin control is a cutting‐edge research of spin optics, which has many applications.^[^ ^36^, ^37^, ^38^ ^]^ Here, we realize successfully a wave‐based XOR logic operation on PDPM. The metasurface‐based XOR logic gate platform has two electric inputs (IN1 and IN2) and a circularly‐polarized (CP) wave readout. The input logical binary digits “0” and “1” of the XOR logic gate are corresponding to the biases *V*_R_ = 0 V (*C*_T_ = 3.20 pF) and *V*_R_ = 20 V (*C*_T_ = 0.35 pF), respectively. The output logical values “0” and “1” indicate two spin states of the output CP waves.

We first analyze theoretically the operation principle of the XOR logic gate for implementing the spin control of CP reflected waves. Assuming that the right‐handed circularly polarized (RCP) wave is incident normally on the XOR logic gate, the incident and reflected electric fields can be expressed as

(1) $$E_{i}=\left(i\hat{x}-\hat{y}\right)E_{m}e^{ikz}$$

(2) $$E_{r}=\left(\hat{x}iR_{x}e^{i\varphi_{x}}-\hat{y}R_{y}e^{i\varphi_{y}}\right)E_{m}e^{-ikz}$$

in which *k* is the wave vector, *R_x_* and *R_y_* are the reflection amplitudes of *x*‐ and *y*‐polarized waves; *φ_x_* and *φ_y_* are the introduced reflection phases by the metasurface under the *x*‐ and *y*‐polarized incidences, respectively. For the four dual‐input states [00], [10], [01], and [11], we know that *R_x_* and *R_y_* are approximately identical (≈1) according to the simulated amplitude responses of the ME. Thus, it is clear from Equation (2) that the spin state of the CP reflected wave depends on the phase difference between *φ_x_* and *φ_y_*. For [00] and [11] inputs, the reflection phases *φ_x_* = *φ_y_*. Thus the spin state of the CP reflected wave is reversed, becoming left‐handed circularly polarized (LCP) wave, because of the inverse propagation direction of the reflected wave. For [10] and [01] inputs, however, the phase difference between *φ_x_* and *φ_y_* equals 180°. Then, Equation (2) can be simplified as

(3) $$E_{r}=\left(\hat{x}-i\hat{y}\right)E_{m}e^{-i\left(kz+\varphi \right)}$$

From Equations (1) and (3), we observe that the CP reflected wave carries the same spin state with the incident wave. Therefore, when the XOR logic gate outputs the digit “0” (false), the spin states of the CP reflected and incident waves are reversed (spin‐reversed), while when the XOR logic gate outputs the digit “1” (true), the spin states of the CP reflected and incident waves are identical (spin‐locked). It should be noted that, although the operation principle of the XOR logic is analyzed and discussed under the RCP incidence, our conclusion is also true for the LCP incidence.

To verify the metasurface‐based XOR logic gate for the spin modulation of CP waves, we have investigated numerically and experimentally the far‐field scattering property of the metasurface. Under the normal incidences of RCP and LCP waves, the normalized far‐field scattering patterns at 5.85 GHz of the XOR logic gate on the *y*–*z* plane are shown in **Figure** **3**. We observe that both simulated and measured results show good XOR logic features for the four inputs of [00], [10], [01], and [11]. In the cases of [00] and [11] inputs, very few scattered CP waves keep the same spin state as the incident CP waves (Figures 3a,d), due to the fact that the spin state of CP waves is reversed upon reflection. On the contrary, for the [10] and [01] inputs, the scattered CP waves have identical spin state as the incident CP waves, and the spin‐locked reflection is well achieved, as shown Figures 3b,c. It is obvious that for the binary output on spin modulations, the power‐level differences of the scattered CP waves between output “0” and “1” states are larger than 30 dB in both simulations and experiments, which indicates that the metasurface‐based XOR logic gate has a strong spin‐modulation depth. The simulated axial ratios (in the +*z*‐direction) of the scattered RCP and LCP waves are below 1 dB at 5.85 GHz for the four input states (see Table S1, Supporting Information), showing high CP purity. All results confirm clearly that the constructed PDPM can well realize dynamic XOR logic operations, which have many practical applications in information and computational technologies. More interestingly, the metasurface‐based XOR logic gate enables fast switching time between the spin‐reversed and spin‐locked states of the output CP waves. Thus digital information can be directly modulated to the spin state of CP waves by using the XOR logic gate, which could provide a solution to rapid polarization‐division multiplexing in high‐speed wireless communication systems.^[^ ^38^, ^39^ ^]^

**Figure 3:**
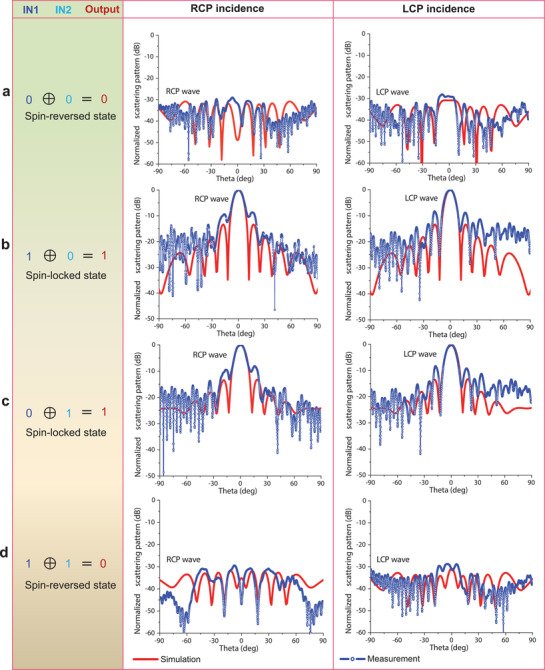
Simulated and measured normalized far‐field scattering patterns at 5.85 GHz of the metasurface‐based XOR logic gate on the *y*–*z* plane. a–d) Performances of the dynamic XOR logic gate with the four inputs of [00], [10], [01], and [11], respectively. All the results confirm that the realized PDPM has good XOR logic features for dynamically controlling the spin states of CP reflected waves.

In the dynamic XOR logic gate, all MEs are controlled simultaneously and have the same digital state. Actually, the 24 input pins in each set of interfaces of PDPM can be actuated independently, which enables high encoding capacity and more complicated functionalities. Here, we realize a fixed‐frequency dual‐beam scanning antenna system in orthogonal directions by the same PDPM prototype. When specific coding sequences are input to PDPM, it will generate two symmetrical tilted beams along the *y*‐ or *x*‐direction under the normal illumination of *x*‐ or *y*‐polarized waves. These two beams can be steered simultaneously at a fixed operating frequency by changing the coding sequences.

For the *x*‐polarized incidence, PDPM can be encoded with different coding sequences varying along the *y*‐direction. As examples, the simulated 3D far‐field radiation beams of the PDPM at 5.85 GHz with four coding sequences S1, S2, S3, and S4 are shown in **Figure** **4**a. We observe that, when the coding sequence changes from S1 to S4, two symmetrical radiation beams in the *y*–*z* plane scan simultaneously, pointing to ±9.6°, ±23.8°, ±36.9°, and ±56.6°, respectively. The scanning angle range can reach as high as 47°. In fact, the beam direction of PDPM can be predicted theoretically by the following equations:^[^ ^22^ ^]^

(4) $$\theta =\arcsin\left(\lambda_{0}\sqrt{\frac{1}{{\Gamma_{x}}^{2}}+\frac{1}{{\Gamma_{y}}^{2}}}\right)$$

(5) $$\phi =\pm \arctan\frac{\Gamma_{x}}{\Gamma_{y}},\;\phi =\pi \pm \arctan\frac{\Gamma_{x}}{\Gamma_{y}}$$

where Γ*_x_* and Γ*_y_* represent the periodic lengths of the coding sequences along the *x‐* and *y*‐directions. Under the *x*‐polarized incidence, the four coding sequences “000000000000111111111111” (S1), “000000111111…” (S2), “00001111…” (S3), and “000111…” (S4) vary along the *y*‐direction. Then the corresponding periodic lengths (Γ*_y_*) are equal to 240, 120, 80, and 60 mm, respectively, while Γ*_x_* is infinity. Considering that *λ*_0_ = 51.3 mm (*λ*_0_ is the free‐space wavelength at 5.85 GHz), the radiation angles are calculated as *θ* = 12.3°, 25.3°, 39.8°, and 58.8°; and *ϕ =* 90° and 270°, which indicate that the two beams are distributed symmetrically in the *y*–*z* plane. It is obvious that the simulated beam angles agree very well with the theoretical predictions with an error smaller than 3°.

**Figure 4:**
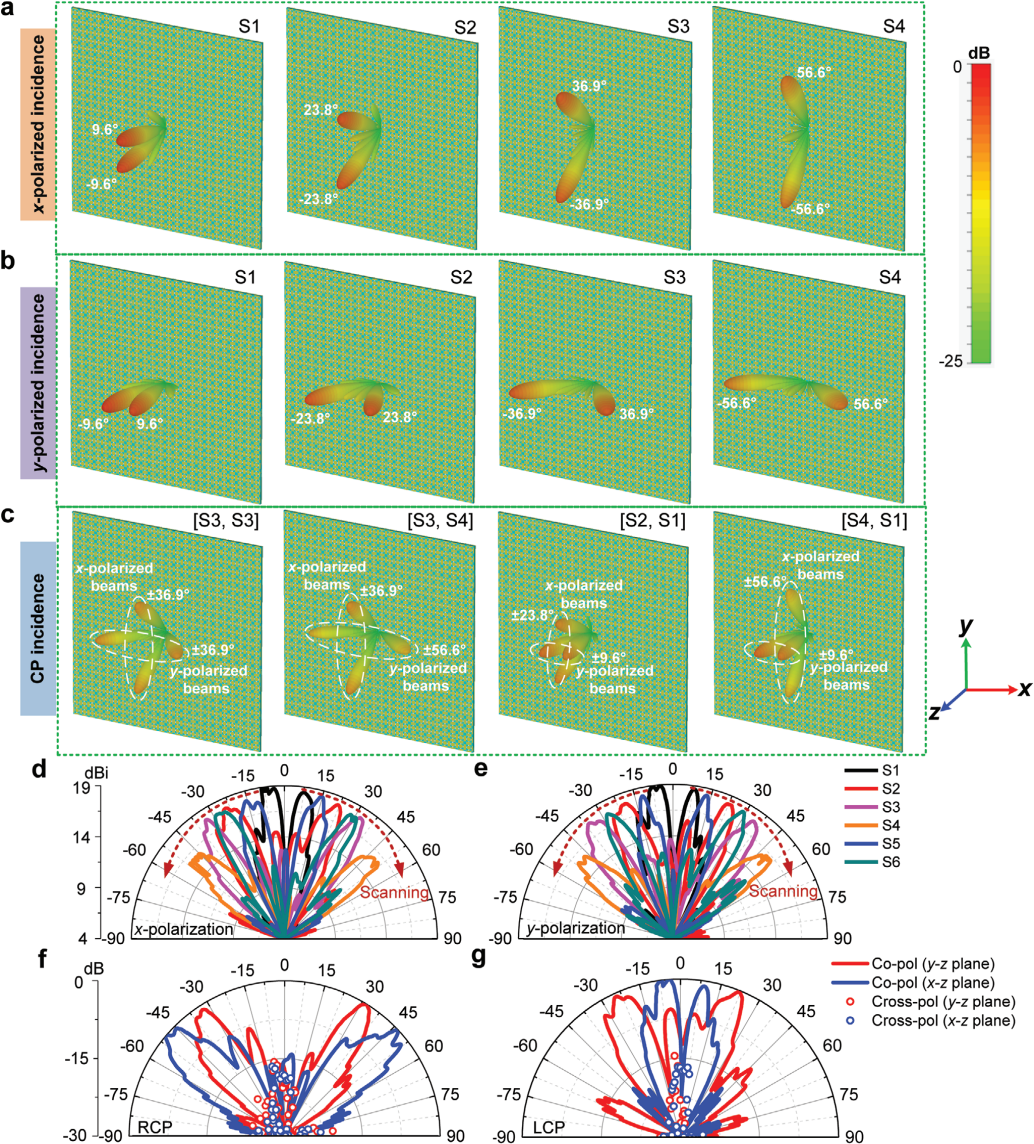
Far‐field performances at 5.85 GHz of the realized dual‐beam scanning antenna and dual‐polarized shared‐aperture antenna systems. a,b) Simulated 3D far‐field radiation patterns of the PDPM with four coding sequences S1, S2, S3, and S4 under the *x*‐ and *y*‐polarized incidences, respectively. c) Simulated 3D far‐field radiation patterns of the PDPM with four dual‐coding configurations under the CP incidence. d,e) Measured 2D gain patterns of the PDPM with the six coding sequences under the *x*‐ and *y*‐polarized incidences, respectively. f,g) Measured 2D gain patterns of the PDPM with two coding configurations [S3, S4] and [S2, S1], respectively.

Similarly, under the *y*‐polarized incidence, PDPM will radiate two symmetrical beams in the *x*–*z* plane. Applying the same coding sequences S1, S2, S3, and S4, which vary along the *x*‐direction, the simulated 3D far‐field radiation beams at 5.85 GHz are illustrated in Figure 4b, in which the two beams are directing at ±9.6°, ±23.8°, ±36.9°, and ±56.6°, respectively, in the *x*–*z* plane. We remark that, beyond the scanning angles discussed above, the same PDPM is capable of generating more different scanning directions when programmed with other coding sequences. For example, under coding sequences “000000001111111100000000” (S5) and “000001111100000111110000” (S6), the two symmetrical radiation beams will appear at ±16.5° and ±29.6°, respectively, as illustrated in Figure S5, Supporting Information.

Under the normal incidence of *x*‐ and *y*‐polarized waves, the measured radiation patterns of the dual‐beam scanning system at 5.85 GHz with the six coding sequences are presented in Figures 4d,e, respectively. We observe that PDPM shows good characteristics of dual‐beam scanning under both polarizations, and the measured two symmetrical beams direct to ±8.5°, ±22.7°, ±34.7°, ±52.3°, ±15.2°, and ±28.6°, respectively, in each case when the coding sequence switches from S1 to S6. The measured peak gain is 18.8 dBi, and the gain variation under the six coding sequences is approximately 3.0 dB. Compared with the traditional beam‐scanning systems based on leaky‐wave antenna, the proposed PDPM has more scanning states, wider scanning angle, and higher gain, and thus can improve the channel capacity, communication quality, and spectrum efficiency, which play a pivotal role in the fifth‐generation (5G) wireless communications.

Since the proposed PDPM is able to realize the dual‐beam scanning in the *x*–*z* and *y*–*z* planes individually under the *y*‐ and *x*‐polarized incidences, it can further work as a dual‐polarized shared‐aperture antenna system with dual‐beam scanning in the upper half space. In this case, the same PDPM operates simultaneously under the *x*‐ and *y*‐polarized waves, and thus can provide two independent information channels at the single frequency. That is to say, PDPM reuses frequency and carries double information capacity, which is particularly important in the era of increasingly scarce spectrum resources. Under the normal incidence of CP waves, PDPM generates polarization‐separating beams, and the dual‐beams of the *x*‐ and *y*‐polarized waves will distribute in the *y*–*z* and *x*–*z* planes, respectively, as shown in Figure 4c. From the first 3D far‐field radiation pattern (under the RCP incidence), we clearly see that the two *x*‐polarized beams and two *y*‐polarized beams (with [S3, S3] coding sequences) are spatially isolated to each other, pointing to ±36.9° in the *y*–*z* and *x*–*z* planes. When the *x*‐ and *y*‐polarized waves are programmed with coding sequences [S3, S4], the scan angles of the *x*‐polarized waves are kept as ±36.9°, while the scan angles of the *y*‐polarized waves are changed to ±56.6°, as illustrated in the second far‐field pattern, showing the capability of independent controls of the *x*‐ and *y*‐polarized waves. Similarly, the proposed PDPM also enables good performance of independent dual‐beam scanning under the LCP incidence, as illustrated in the third and fourth far‐field patterns under the coding sequences of [S2, S1] and [S4, S1].

For experimental verification, we just report the measured 2D radiation patterns corresponding to the second and third cases in Figure 4c. Under the RCP incidence, when the control interfaces I and II of PDPM are programmed with coding sequences [S3, S4], we observe four obvious pencil beams, in which the two *x*‐polarized beams (co‐polarization in the *y*–*z* plane) are directed to ±34.3°, and the two *y*‐polarized beams (co‐polarization in the *x*–*z* plane) are directed ±52.1°, as shown in Figure 4f. Under the LCP incidence, when the control interfaces I and II of PDPM are programmed with coding sequences [S2, S1], the radiated two groups of polarization‐separating beams are pointed to ±8.5° and ±22.8°, respectively, as demonstrated in Figure 4g. In the main beam areas, the measured cross‐polarization is approximately 15 dB lower than the co‐polarization peak, indicating that the PDPM system has high cross‐polarization isolation.

In summary, we demonstrated a dual‐programmable metasurface with high‐stability and rich rewritability. The proposed PDPM was implemented by integrating varactors in an ingeniously‐designed ME, which can be programmed with two independent coding sequences simultaneously through the control circuits, thus enabling powerful ability in controlling the *x*‐ and *y*‐polarized waves independently. Compared to the previous programmable metasurfaces, PDPM has more degrees of freedom to greatly enhance the EM functionalities of metasurface‐based devices and systems. We fabricated a PDPM prototype, from which three completely different devices with programmable functions were realized using the same metasurface. The first is an XOR logic gate information platform for dynamically controlling the spin state of CP waves, the second is a dual‐beam scanning antenna system, and the third one is a dual‐polarized shared‐aperture antenna system. The dual‐polarization operating feature of PDPM on the one hand further improves the integration level of the metasurface‐based electronic systems, on the other hand increases the capability to control the EM waves and digital information. The realized PDPM system has good performance in terms of wide angle scanning, independent dual‐plane scanning, high gain, and high cross‐polarization isolation. Besides the several demonstrated features, PDPM can also realize many other functions, such as dual‐polarization independent cloaking and illusion and dynamic polarization conversions. The dual‐programmable metasurface not only sets up a bridge between the subwavelength‐scale light–matter interaction and the information processing, but also provides an alternative route to realize large‐scale dynamic meta‐devices and software‐defined rewritable systems, which is fundamentally important to new computing and communications paradigms.^[^ ^40^, ^41^ ^]^

## Experimental Section

##### Design of Extended Interface Circuit

In the control circuits, the FPGA controller provided 12‐way control signals: an enable signal, 3‐way address signals, and 8‐way data signals, which were used to control the extended interface circuit. The fabricated interface circuit consisted of a “74HC238” decoder chip and six “74HC373” D‐type latch chips. The enable signal and 3‐way address signals were directly input to the decoder, and the 8‐way data signals were imported to the input ports of every D‐type latch chip in parallel. Additionally, the six output terminals of the decoder were connected to the enable pins of six latches, respectively (see Figure S3, Supporting Information). With this configuration, the six outputs of the decoder were all low level when the enable signal was low level, and thus the output states of the six latch chips would be unchanged. That is to say, when the connection between FPGA and interface module was interrupted, it would lead to a low level of the enable signal. Then all latches would maintain their original output states, and thus PDPM could still work steadily. On the contrary, when the enable signal was in high level, the decoder would enable one of the six latches according to the value of 3‐way address signals, and then the 8‐way data signals from FPGA would be transmitted to the eight output ports of the latch. Because the response's speeds of the “74HC238” decoder chip and “74HC373” latch chip were 16 and 12 ns, respectively, the switching time of each operation cycle was about 28 ns. To update the status of all 48 output pins, six operation cycles were required. Thus, the total switching time of the interface module was around 168 ns. The power consumption of the “74HC238” decoder and the “74HC373” latch were approximately 36.1 and 2.9 mW, respectively.

##### Design of Voltage Conversion Circuit

To convert the low pin‐voltage to the required 20 V voltage for realizing the “1” element of PDPM, a bipolar transistor DC voltage conversion circuit was designed, as shown in Figure S4a, Supporting Information. The base, collector, and emitter of the “S8050‐J3Y” bipolar transistor were connected to one output pin of the aforementioned latch (by a 470 Ω current‐limiting resistor), a 20 V external voltage source (by a 10 kΩ pull‐up resistor), and the ground, respectively. Moreover, the collector also worked as the output of the DC voltage conversion circuit. When the input voltage was 3.3 V, the bipolar transistor operated in a saturation state, resulting in conduction between the collector and emitter, and thus the output voltage of the bipolar transistor was approximately 0 V (see Figure S4b, Supporting Information). When the input voltage was 0 V, the bipolar transistor worked in a cutoff state, and the output voltage was 20 V (see Figure S4c, Supporting Information). The designed DC voltage conversion circuit had good conversion performance and the switching speed of the “S8050‐J3Y” bipolar transistor was about 7 ns. Hence, the switching speed of the realized control circuits was very fast (in nanosecond level).

##### Sample Fabrication

The metasurface sample was fabricated by low‐cost printed circuit board technology, and then the 2400 miniature “Skyworks SMV2020‐079LF” varactor chips were integrated precisely in the passive metasurface through the well‐developed machine welding procedure. The fabricated PDPM sample totally covered an area of 260 × 260 mm^2^. The total thickness of the ultrathin PDPM sample was 3.37 mm (nearly 0.066 *λ*_0_).

##### Experimental Setup

To avoid interference from the environment, all the far‐field experiments were carried out in a microwave chamber. In measurement, both the PDPM sample and the transmitting antenna were placed on a supporting board, and the distance between them was approximately 1.6 m. The supporting board with similar EM property to the air was fixed on an automatic antenna turntable that could be controlled remotely by a computer and could be rotated continuously with high precision in the horizontal plane (scanning 360°). The experimental setup is shown in Figure S6, Supporting Information. Two identical dual‐circular‐polarized conical horn antennas were adopted for generating and receiving CP waves, respectively. Additionally, the standard gain horn antenna with gain of 15 dBi and operating bandwidth from 5.38 to 8.17 GHz was used to emit linearly‐polarized quasi‐plane wave illumination. When the supporting board carrying the transmitting antenna and the PDPM sample rotated from ‐90° to 90°, the receiving antenna (1–18 GHz double‐ridged horn antenna) received the reflected linearly‐polarized waves in the horizontal plane. It should be noted that, the electric fields distributed in the horizontal plane due to the fact that the turntable can only rotate in the horizontal plane can only be monitored. Therefore, to measure the linearly‐polarized beams appearing in vertical plane, the PDPM sample should be revolved 90°. All the coding sequences were generated by the control circuits and were input into the PDPM sample through its two sets of control interfaces I and II.

## Conflict of Interest

The authors declare no conflict of interest.

## Supporting information

Supporting Information
