# Supplementary material for: Polarization‐Controlled Dual‐Programmable Metasurfaces
Source: Adv Sci (Weinh). 2020 Apr 16;7(11):1903382. doi: 10.1002/advs.201903382 (PMC7284210; doi:10.1002/advs.201903382)
Supplement: Supplementary file 1 — Supporting Information [file ADVS-7-1903382-s001.pdf]

Supporting Information

Polarization-controlled dual-programmable metasurfaces

Xin Ge Zhang, Qian Yu, Wei Xiang Jiang, Ya Lun Sun, Lin Bai, Qiang Wang, Cheng-Wei Qiu* and Tie Jun Cui**

Bias network design of ME. The two rectangular patches in the *x*-direction are connected to the bias line I through two metallic vias, and the other two rectangular patches in the *y*-direction are connected to the bias line II by the other two metallic vias. Bias lines I and II are etched on the top and bottom of the thin F4B substrate, respectively, which is attached to the back of the resonator by a 0.12-mm-thick FR4 (ε_r = 4.3, tanδ = 0.025) adhesive layer. Hence, the resonator and DC bias network are well integrated to form a dual-programmable ME. In addition, the central square patch is connected to the metal ground through a metallic via. We arrange bias lines I and II in the *x*- and *y*-directions, respectively.

Structure parameters of ME. The sidelength of the element is 10.0 mm, which is around 1/5 wavelengths at 5.85 GHz. All the copper sheets are 18-um-thick, and the thickness of substrate of the resonator is 3.0 mm. It should be noted that when the *x*- and *y*-polarized EM waves are normally incident to the element, the resonance currents will flow through the bias lines I and II, respectively, through the corresponding metallic vias, as shown in **Figure S1**. Because the bias lines I and II are on top and bottom of the substrate with a certain thickness, respectively, the two current paths are different. Thus, even under the same capacitance configuration, the element will generate different resonance responses for the two orthogonal polarization states due to the electrical asymmetry of the DC bias network. To minimize the difference, we select the ultrathin F4B dielectric with 0.25 mm thickness as the DC bias layer, and the widths of the two bias lines I and II are set as different values. The diameters of all five metallic vias are 0.5 mm. The lengths and widths of two metal pads in each 1.5-mm-

width gap are 0.5 and 0.3 mm, respectively. In the simulation, since there is no existing varactor model in the commercial software, CST Microwave Studio, we use the RLC series circuit consisting of a resistor (R_S = 2.5 Ω), an inductor (L_S = 0.7 nH), and an adjustable capacitor (variable C_T) to mimic the embedded "Skyworks SMV2020-079LF" varactor. To achieve the required reflection performance, we do a great deal of numerical simulations on the element, and the key parameters are optimally set as L_1 = 3.8 mm, L_2 = 1.5 mm, L_3 = 6.4 mm, W_1 = 0.6 mm, and W_2 = 3.0 mm, respectively.

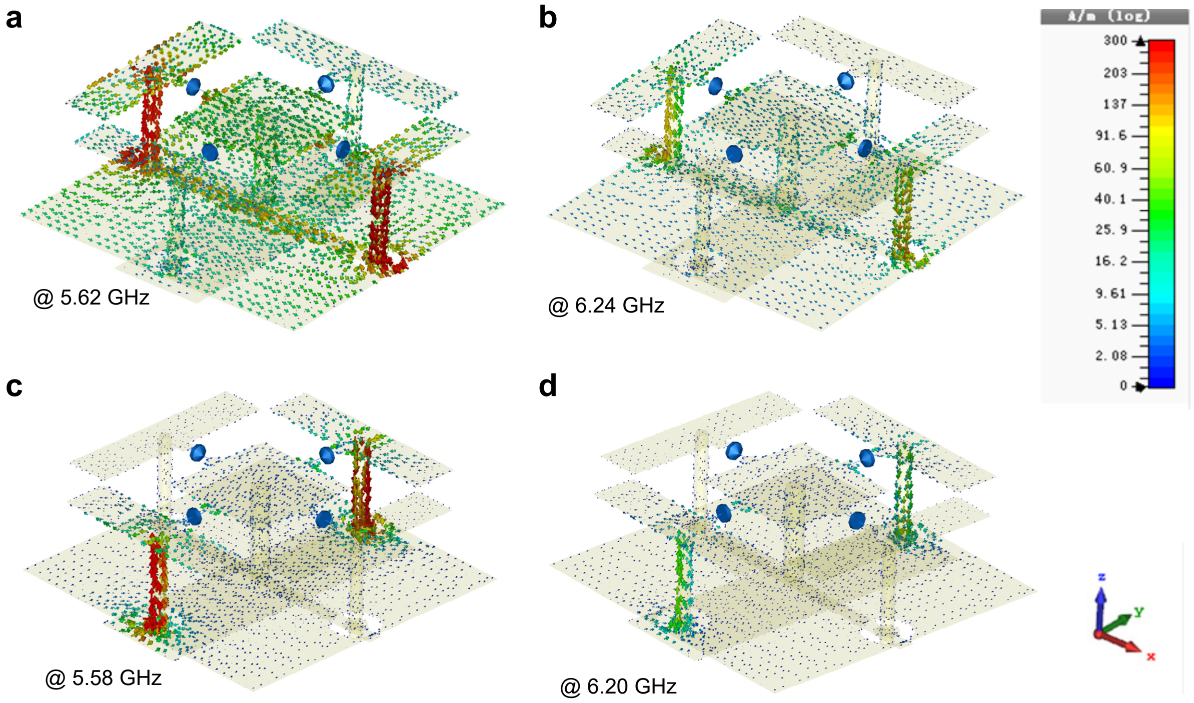

Figure S1. Simulated induced surface-current distributions of the designed dual-programmable ME at corresponding resonance frequencies. a,b) Distributions of induced surface currents of the ME for C_{Tx} = 3.20 pF and C_{Tx} = 0.35 pF, respectively. We observe that the resonance currents flow through the bias line I at 5.62 and 6.24 GHz under *x*-polarized incidence. c,d) Distributions of induced surface currents of the ME for C_{Ty} = 3.20 pF and C_{Ty} = 0.35 pF, respectively. We see clearly that the resonance currents flow through the bias line II at 5.58 and 6.20 GHz under *y*-polarized incidence.

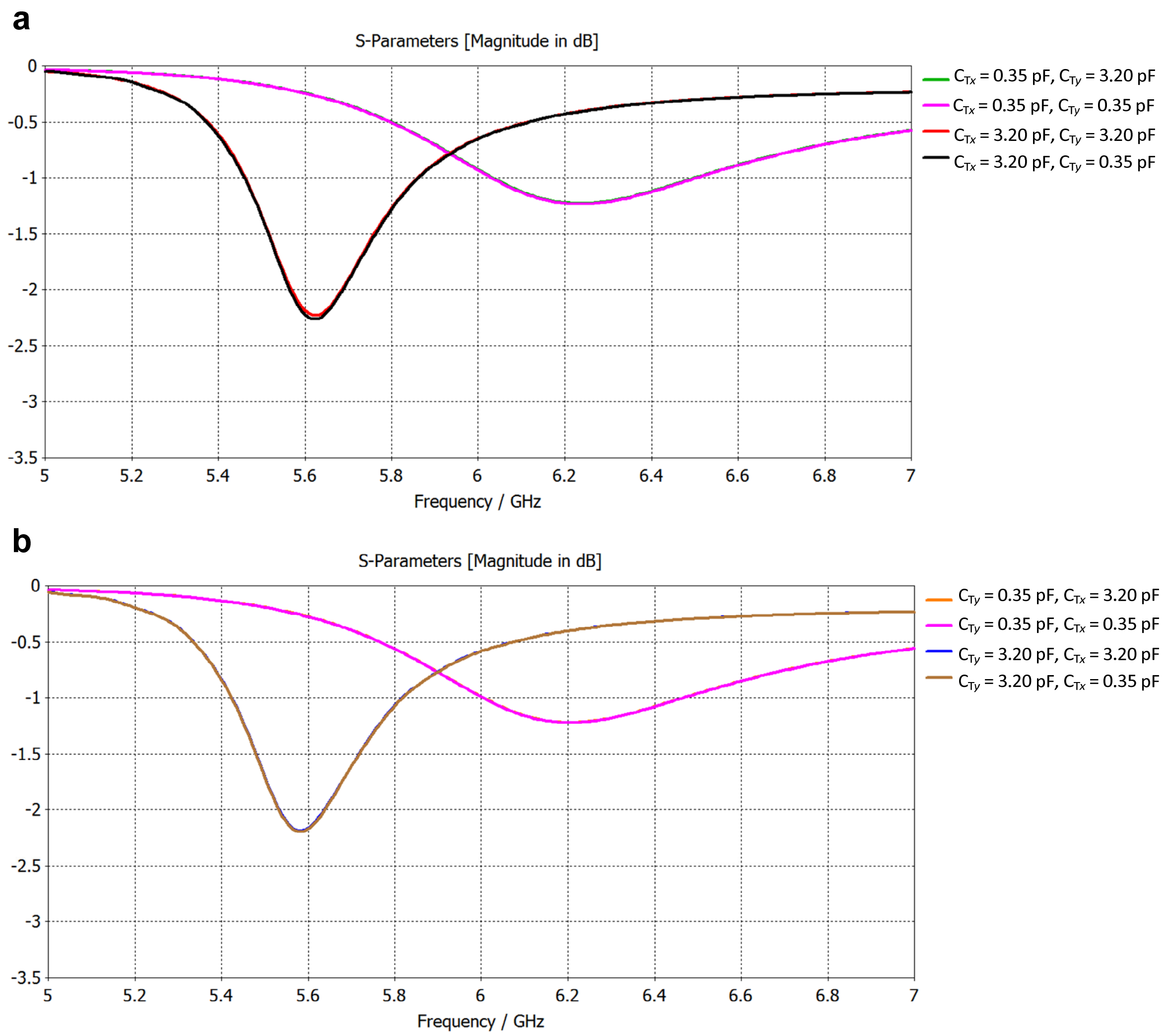

Figure S2. a,b) Simulated reflection amplitudes of the programmable ME with different capacitance configurations under *x*- and *y*-polarized incidences, respectively. It is obvious that when *x*- or *y*-polarized EM waves propagates normally into the ME, changing the capacitance of the two varactors in EM-wave cross-polarization direction has no effect on the resonance response, which indicates that the realized ME has high polarization stability and cross-polarization isolation.

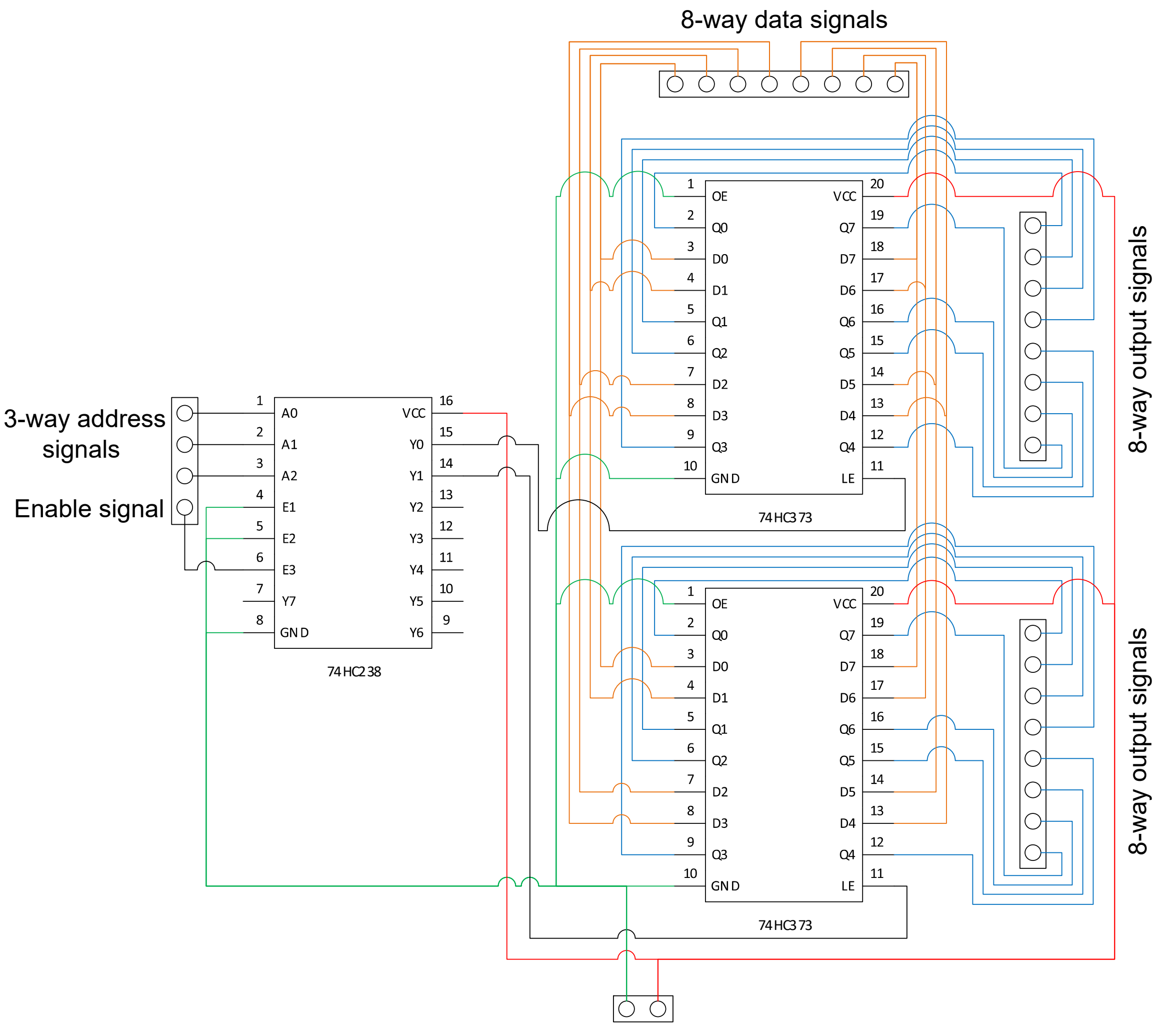

Figure S3. Detailed circuit schematic of the designed extended interface circuit. The 6 output signals (Y0-Y6) of the decoder are connected to the enable pins (LE) of the six latches, respectively, for controlling the state of the six latches. Each latch can generate 8-way independent output signals to actuate the dual-programmable metasurface. Here, for the sake of brevity, we just show two latches in this circuit schematic. The connection rules of other four latches are similar to those of the two latches.

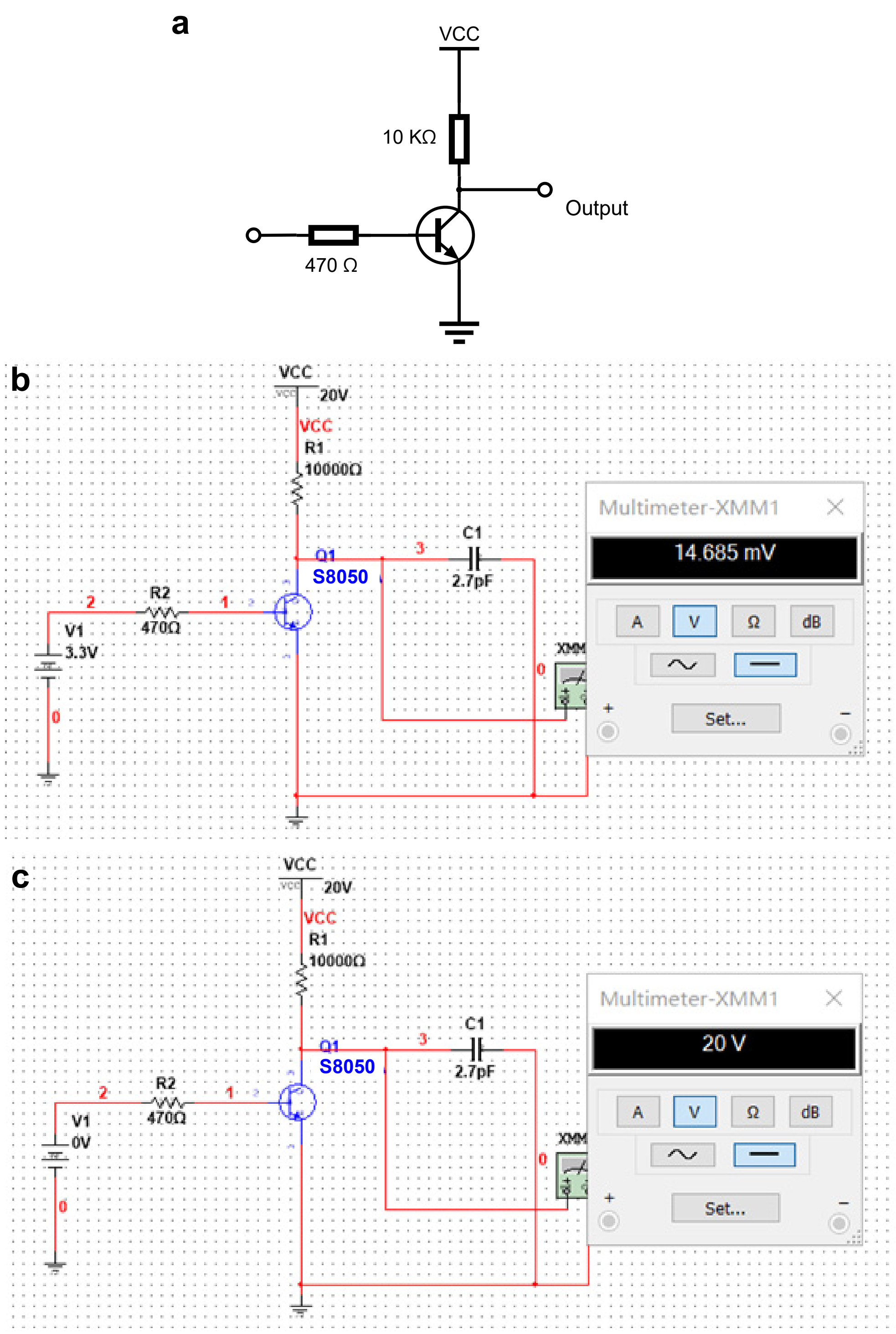

Figure S4. a) A bipolar transistor DC voltage conversion circuit. b,c) Simulated output voltages of the DC voltage conversion circuit for input voltages of 3.3 and 0 V, respectively. From the simulation results, we observe that the DC voltage conversion circuit can generate two required voltages of 0 and 20 V for the "Skyworks SMV2020-079LF" varactor.

Table S1. Simulated axial ratios (in +*z*-direction) of the realized XOR logic gate at 5.85 GHz for the four dual-inputs [00], [10], [01] and [11].

**IN1**	**IN2**	**Output**	**Axial ratio (dB)**	
			RCP incidence	LCP incidence
0	0	0	0.12	0.14
1	0	1	0.94	0.96
0	1	1	0.82	0.88
1	1	0	0.43	0.32

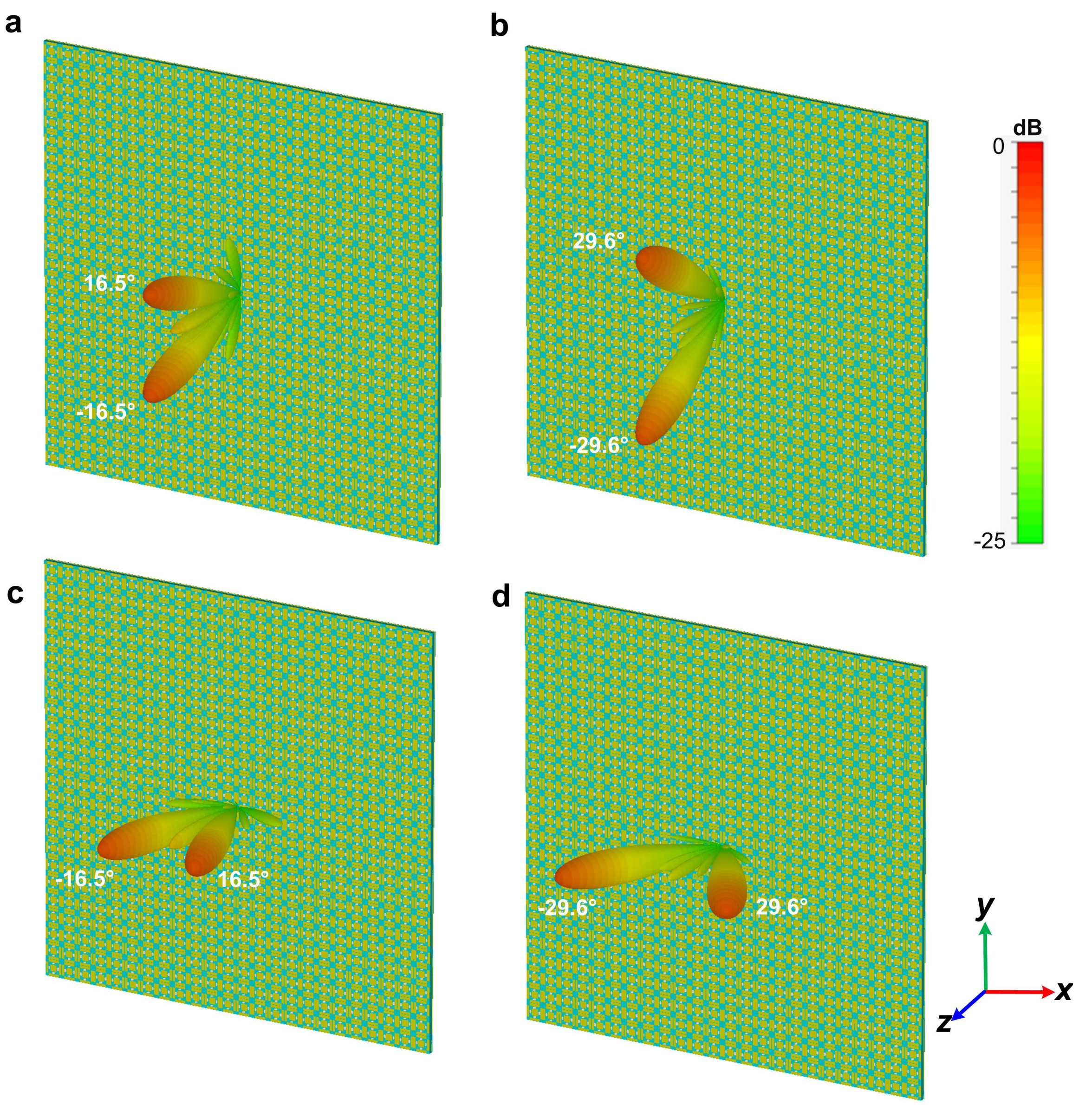

Figure S5. a,b) Under the *x*-polarized incidence, simulated 3D far-field radiation patterns at 5.85 GHz of the dual-programmable metasurface with two coding sequences S5 and S6, respectively. c,d) Under the *y*-polarized incidence, simulated 3D far-field radiation patterns at 5.85 GHz of the dual-programmable metasurface with two coding sequences S5 and S6, respectively.

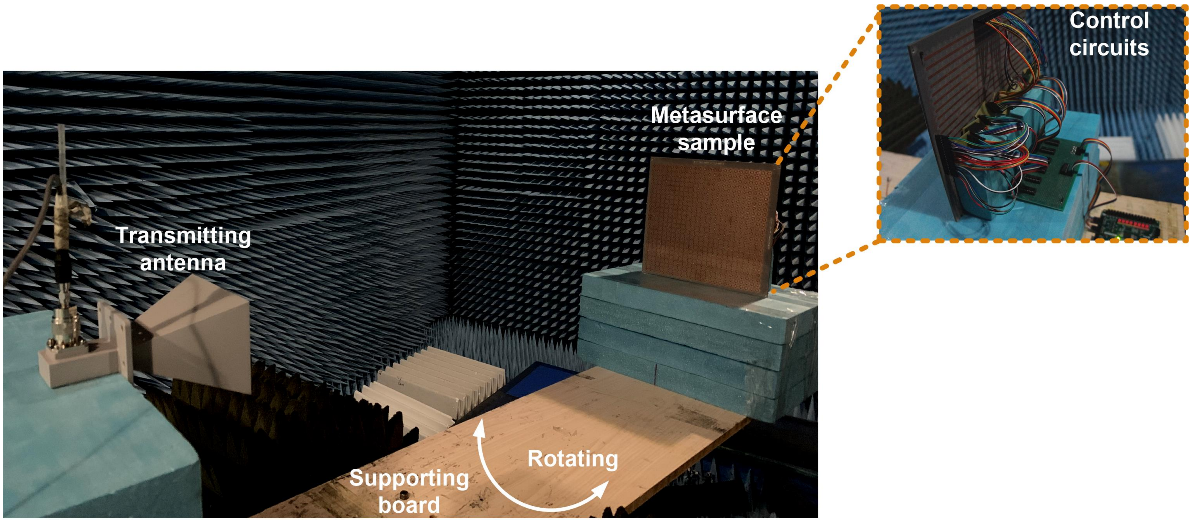

Figure S6. Experimental setup in an anechoic chamber. The inset shows details of the control circuits of the dual-programmable metasurface sample.
